# Caenorhabditis elegans egg-laying and brood-size changes upon exposure to Serratia marcescens and Staphylococcus epidermidis are independent of DBL-1 signaling

**DOI:** 10.17912/2r51-b476

**Published:** 2019-04-15

**Authors:** Bhoomi J Madhu, Aileen E Salazar, Tina L Gumienny

**Affiliations:** 1 Department of Biology, Texas Woman's University, Denton, TX, 76204-5799

**Figure 1.**
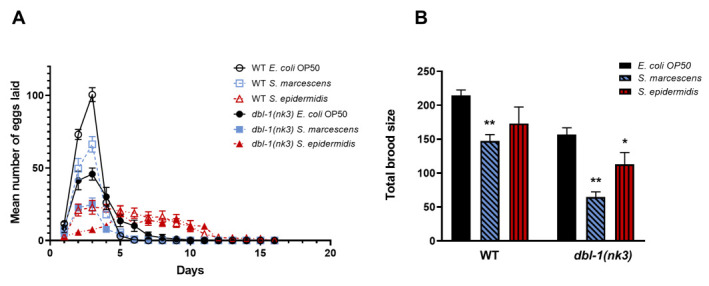
Effects of *S. marcescens* and *S. epidermidis* on egg laying and brood size in wild-type and *dbl-1(nk3)* populations. (A) Comparison of mean eggs laid between wild-type (WT) and *dbl-1(nk3)* populations fed on *E. coli* OP50, *S. marcescens*, or *S. epidermidis*. The counts of number of eggs laid were reported only for plates with live animals. Error bars represent SEM. n = 7-10 surviving animals each. (B) Comparison of total brood size between wild-type and *dbl-1(nk3)* populations fed on *E. coli* OP50, *S. marcescens*, or *S. epidermidis*. Total brood sizes of animals that desiccated were censored. Error bars represent SEM. n = 7-10 surviving animals each. Within each genetic background, significant differences between the *E. coli* OP50-fed control and the pathogen-fed populations are marked with asterisks (*, *p*< 0.05, **, *p*< 0.001).

## Description

*Caenorhabditis elegans* naturally thrives in a soil environment where they feed on bacteria and are in constant association with a diverse range of microbes (Barker *et al.* 1994). *C. elegans* egg laying is delayed or reduced when animals are infected with *Burkholderia pseudomallei*, *Burkholderia thailandensis*, *Staphylococcus aureus*, and *Serratia marcescens* (Mallo *et al.* 2002; O’Quinn *et al.* 2002; Irazoqui *et al.* 2010). These changes in egg laying may be a protective response to pathogenic bacteria. Mutants of TGF-β-like DBL-1 signaling pathway also display reduced brood size (Luo *et al.* 2009; Roberts *et al.* 2010). While the peak of egg-laying activity seen in normal animals between days two and four is depressed in *dbl-1* pathway mutants, the reproductive span of these *dbl-1* pathway mutants is increased to up to 13 days (Luo *et al.* 2009). To determine if the egg-laying observed during infection is DBL-1 pathway-dependent, we tested the effect of the DBL-1 signaling pathway on egg laying when *C. elegans* were fed on representative Gram-negative (*S. marcescens*) and Gram-positive bacteria (*Staphylococcus epidermidis*).

Similar to previously published reports, we found that loss of DBL-1 pathway signaling decreases brood size and increases reproductive span in normal laboratory conditions (*E. coli* strain OP50 and 20°C incubation) (Figure 1A and B) (Luo *et al.* 2009; Roberts *et al.* 2010).

Here, we report three new results. First, brood size reductions caused by infection and by loss of DBL-1 signaling are independent (Figure 1A). Wild-type and *dbl-1(nk3)* animals both significantly decrease their brood size when grown on *S. marcescens* (*p*= 0.005 and *p*< 0.001, respectively). *dbl-1* mutant animals laid even fewer eggs than the wild-type animals on *S. marcescens*, suggesting that the reduced brood size phenotype is independently affected by both *S. marcescens* exposure and by loss of DBL-1 (*p*< 0.001). While the decrease in brood size of wild-type animals on *S. epidermidis* was not significant (*p*= 0.115), the decreased brood size of *dbl-1(nk3)* animals was significant on this pathogenic bacterial strain (*p*= 0.045). Indeed, the decreases in brood size upon infection with either *S. marcescens* or *S. epidermidis* in both wild-type and *dbl-1(nk3)* populations are similar (*p*= 0.57), suggesting the pathogenic bacteria affect brood size independent of DBL-1. Because *dbl-1(nk3)* populations display a further reduced brood size upon infection by either pathogen compared to the wild type, the negative effects of pathogen exposure and loss of DBL-1 signaling on brood size appear to be additive (*p*< 0.05).

Second, while the wild-type population on *S. marcescens* survived until all animals ceased laying eggs, all *dbl-1(nk3)* animals died on *S. marcescens* by Day 5. These results explain why the extended reproductive span normally seen in *dbl-1(nk3)* populations was not observed on *S. marcescens*. These results also support previous reports of decreased viability of *dbl-1* mutant animals on another variety of *S. marcescens*, Db11 (Mallo *et al.* 2002).

Third, *S. epidermidis* affects egg-laying patterns similar to loss of *dbl-1* function. Initially, both wild-type and *dbl-1(nk3)* strains on *S. epidermidis* have reduced eggs laid in the first four days compared to strains grown on the *E. coli* control. Loss of DBL-1 further reduced the number of eggs laid during each of these days, suggesting that this phenotype is independently affected by both *S. epidermidis* exposure and by loss of DBL-1 (*p*= 0.004). Then, both wild-type and *dbl-1(nk3)* strains on *S. epidermidis* have similar extended reproductive spans, extending to at least day 13 (one tenacious wild-type hermaphrodite laid embryos until day 15). This *S. epidermidis*-induced reproductive span extension appears to be independent of DBL-1 signaling, because the numbers of eggs laid by both wild-type and *dbl-1(nk3)* populations between days 5 and 16 were similar at these time points (*p*= 0.509).

## Methods

Animals were age-synchronized by hypochlorite treatment (Stiernagle, 2006) and grown on plates seeded with *Escherichia coli* OP50. Ten L4 animals were manually transferred to individual plates seeded with *S. marcescens* or *S. epidermidis*. Plates were completely covered by bacteria to prevent animals from avoiding the bacteria. Adults were daily transferred to new plates and the number of eggs laid on each plate was counted every 24 hours until no more eggs were laid. Statistical analyses were performed using repeated measures ANOVA and Tukey’s post-hoc test.

## Reagents

Strains were maintained on EZ media plates at 20°C (0.55 g Tris-Cl, 0.24 g Tris base, 3.1 g BD Bacto^TM^ Peptone, 8 mg cholesterol, 2 g sodium chloride, 20 g agar, in water to 1 L (E. Lambie, personal communication). The *C. elegans* strains used were N2 and NU3 *dbl-1(nk3).* The Gram-negative bacterial strains used were *Escherichia coli* OP50 (CGC) and *Serratia marcescens* (Carolina Biological Supply Company). The Gram-positive bacterial strain used was *Staphylococcus epidermidis* (ATCC 49134). *S. marcescens* and *S. epidermidis* were provided by A. J. Hammett, TWU. All bacterial strains were grown for 9 hours in tryptic soy broth at 37°C before plating on EZ media plates.
